# A comparison of carbon ion radiotherapy and transarterial chemoembolization treatment outcomes for single hepatocellular carcinoma: a propensity score matching study

**DOI:** 10.1186/s13014-019-1347-4

**Published:** 2019-08-02

**Authors:** Shintaro Shiba, Kei Shibuya, Hiroyuki Katoh, Takuya Kaminuma, Masaya Miyazaki, Satoru Kakizaki, Ken Shirabe, Tatsuya Ohno, Takashi Nakano

**Affiliations:** 1 Department of Radiation Oncology, Gunma University Graduate School of Medicine, 3-39-22, Syowa-machi, Maebashi, Gunma 371-8511 Japan; 2 Gunma University Heavy Ion Medical Center, 3-39-22, Syowa-machi, Maebashi, Gunma 371-8511 Japan; 3 Department of Radiation Oncology, Kanagawa Cancer Center, 2-3-2, Nakao, Asahi-ku, Yokohama, Kanagawa 241-8515 Japan; 4 Department of Applied Medical Imaging, Gunma University Graduate School of Medicine, 3-39-15 Showa-machi, Maebashi, Gunma 371-8511 Japan; 5 Department of Gastroenterology and Hepatology Science, Gunma University Graduate School of Medicine, 3-39-15 Showa-machi, Maebashi, Gunma 371-8511 Japan; 6 Department of Hepatobiliary and Pancreatic Surgery, Gunma University Graduate School of Medicine, 3-39-22, Syowa-machi, Maebashi, Gunma 371-8511 Japan

**Keywords:** Carbon ion radiotherapy, Hepatocellular carcinoma, Transarterial chemoembolization, Propensity score matching

## Abstract

**Background:**

We compared clinical outcomes of carbon ion radiotherapy and transarterial chemoembolization in the treatment of hepatocellular carcinoma.

**Methods:**

Data of 477 patients with hepatocellular carcinoma who had undergone carbon ion radiotherapy or transarterial chemoembolization between April 2007 and September 2016 were retrospectively reviewed. Treatment naïve patients with single HCC, who underwent carbon ion radiotherapy or transarterial chemoembolization as a primary treatment were included. Clinical outcomes of the treatments were compared after utilizing propensity score matching.

**Results:**

Of 124 patients who received carbon ion radiotherapy and 353 patients who received transarterial chemoembolization, 31 and 23 patients met our inclusion criteria, respectively. After utilizing propensity score matching, 17 matched pairs of patients from each treatment group were analyzed. The median follow-up durations after carbon ion radiotherapy and transarterial chemoembolization were 43 and 32 months, respectively. The 3-year overall survival, local control, and progression-free survival rates in the carbon ion radiotherapy versus transarterial chemoembolization groups were 88% versus 58% (*p* < 0.05), 80% versus 26% (*p* < 0.01), and 51% versus 15% (*p* < 0.05), respectively.

**Conclusions:**

Carbon ion radiotherapy showed more favorable clinical outcomes than did transarterial chemoembolization for patients with single hepatocellular carcinoma after matching patient characteristics utilizing propensity score matching. Further studies with larger patient numbers are required to confirm our results.

**Trial registration:**

UMIN000036455: date of registration 22 March 2019, retrospectively registered.

## Background

There are several treatment options for hepatocellular carcinoma (HCC) such as surgical resection, liver transplantation, percutaneous radiofrequency ablation (RFA), transarterial chemoembolization (TACE), molecular targeting therapy, and radiotherapy. Treatment decisions for HCC should include patient-related and disease-related factors.

TACE is the standard treatment for patients with Barcelona Clinic Liver Cancer (BCLC) stage B HCC, and TACE is considered one of the standard treatment options for patients with BCLC stage A who are ineligible for surgery and/or RFA [1, 2]. Several studies have reported that 3-year overall survival (OS) rates for patients treated with TACE for HCC were between 26 and 65% and that 2-year local control (LC) rates were between 28 and 41% [3–9].

Radiotherapy, including stereotactic body radiotherapy, proton beam therapy, and carbon ion radiotherapy (C-ion RT), is a locoregional treatment method for HCC. The use of radiotherapy for HCC has been limited due to poor radiation tolerance of the healthy liver. On the other hand, C-ion RT can reduce healthy liver damage because of its superior dose distribution properties due to a distal tail-off of the Bragg’s peak and a sharp lateral penumbra, which is characteristic of C-ion RT [10]. C-ion RT has been used as a form of therapy for localized HCC patients who are ineligible for surgery and/or RFA, and for patients who decline surgery and/or RFA, where such patients have usually been treated with TACE according to various guidelines [1, 11]. Several studies have reported encouraging clinical outcomes, especially for patients with high rates of LC (3-year LC rates; 81–96%) after C-ion RT for HCC [12–17].

Recently, propensity score matching (PSM) has been used to compare the clinical outcomes of two different treatment modalities in retrospective analyses [5, 18–21]. PSM mimics some characteristics of a randomized controlled trial and has been proposed as a method to minimize potential selection bias of patients in one retrospective non-randomized study [22]; therefore, PSM was adopted to compare the clinical outcomes of two different treatment modalities. To date, there have been no comparative studies of the clinical outcomes between C-ion RT and TACE for single HCC as a primary treatment utilizing PSM. Hence, we conducted a retrospective study comparing the clinical outcomes of C-ion RT and TACE for single HCC as a primary treatment utilizing PSM.

## Methods

### Patients

In this retrospective analysis, we reviewed the medical records of 477 patients treated with C-ion RT or TACE for HCC at Gunma University between April 2007 and September 2016. HCC in all patients had been confirmed according to histology or to the typical hallmarks of HCC, using radiological four-phase multidetector-row computed tomography (CT) imaging or dynamic contrast-enhanced magnetic resonance imaging (MRI) (hypervascular in arterial phase with washout in portal venous or delayed phases). Patients were eligible for this study if they had treatment naïve single HCC with 1) no direct infiltration of the gastrointestinal tract; 2) absence of intrahepatic metastasis; 3) absence of distant metastasis, and; 4) no major vascular invasion, and had received C-ion RT or TACE as a primary treatment. If patients had received C-ion RT or TACE combined with RFA, they were excluded from the study. The Child-Pugh score and class were calculated to evaluate liver function in all patients. The disease stage, according to the BCLC classification, [2] was determined using CT, MRI, ultrasonography, and other relevant variables. This study complied with the standards of the Declaration of Helsinki and current ethical guidelines and was reviewed and approved by the Institutional Review Board (approval number HS2018–271).

### Carbon ion radiotherapy

Immobilization devices including tailor-made fixation cushions and thermoplastic shells were fabricated for patients, and a treatment-planning CT of respiratory-gated CT and four-dimensional CT (4-D CT) images were then acquired. Contrast-enhanced CT images were also taken simultaneously and merged with treatment planning CT images to precisely delineate the gross tumor volume (GTV). We defined the clinical target volume (CTV) margin as the GTV plus 5 mm in all directions, including microscopic disease progression. The planning target volume (PTV) was defined as a summation of the CTV, and the internal margin was defined as the extent of tumor motion shown in the 4-D CT images and the setup margin. We used XiO-N (version 4.47; Elekta AB, Stockholm, Sweden and Mitsubishi Electric, Tokyo, Japan), which has been previously used for treatment planning [23]. The radiation dose calculation for the target volume and the surrounding normal structures was expressed in Gy [relative biologic effectiveness (RBE)], which is defined as the physical dose multiplied by the RBE of carbon ions [10, 24].

**Fig. 1:**
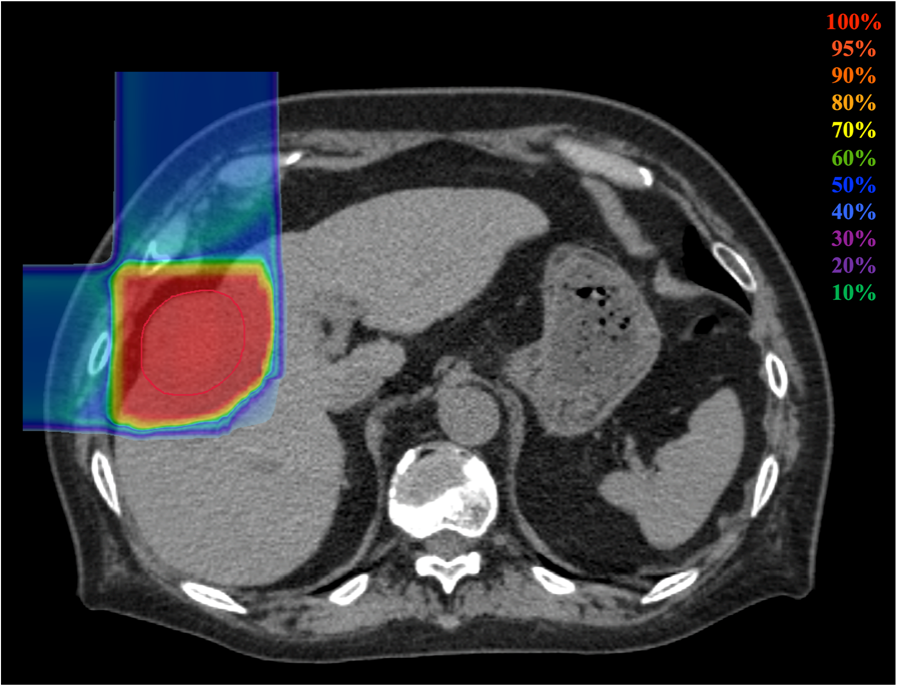
Dose distribution of C-ion RT for HCC. Isodose curves of C-ion RT are superimposed on an axial computed tomography image for the total irradiation plan. The area within the red outline is the gross target volume. Highlighted are 100% (red), 95% (light red), 90% (orange), 80% (light orange), 70% (yellow), 60% (green), 50% (blue), 40% (cyan), 30% (light purple), 20% (purple), 10% (light blue) isodose curves (100% was 60 Gy [relative biological effectiveness])

Prescribed doses were 52.8 Gy (RBE) or 60.0 Gy (RBE) in 4 fractions for standard cases and 60.0 Gy (RBE) in 12 fractions for close-to-gastrointestinal-tract cases. The planning aim was to cover the PTV with at least 95% of the prescribed dose. Dose constraints were as follows: 1) D_1cc_ < 40 Gy (RBE) to the gastrointestinal tract and 2) V_20_ < 35% to the liver [25, 26]. The dose to the portal vein and bile duct was reduced as much as possible. Figure 1 shows a typical radiation field with dose distribution.

Patients received C-ion RT once daily, 4 days per week (Tuesday to Friday). For daily patient position matching, a fiducial gold marker was inserted into the liver. Patient positioning with fiducial marker matching was confirmed using digital orthogonal radiograph images and reference images, which were digitally reconstructed based on CT images for treatment planning [27].

### Transarterial chemoembolization

Selective hepatic arteriography was performed by interventional radiologists using standard and coaxial angiographic techniques via a transfemoral arterial approach with 1.9- to 2.5-French microcatheter and micro-wires for tumor-feeding arterial branches. Whenever possible, super-selective TACE, in which the catheter is additionally advanced into the sub-segmental branches feeding the tumor, was attempted [28]. After microcatheter placement, a mixture of miriplatin or epirubicin with ethiodized oil and gelatin sponge particles was injected. Chemoembolization was performed to complete vessel occlusion and stasis in all patients. Patients were admitted overnight for routine supportive care including intravenous hydration and prophylactic antibiotics.

### Evaluation during follow-up

After completion of C-ion RT or TACE, the patients were followed up with routine blood cell counts, blood chemistry testing, and abdominal diagnostic imaging such as four-phase multidetector-row CT, dynamic contrast-enhanced MRI, or contrast-enhanced ultrasonography. Child-Pugh class progression was evaluated in terms of liver function toxicity status. Local recurrence was defined as tumor regrowth with enhancement of the contrast effect on CT, or MRI, or ultrasonography in the irradiated field after C-ion RT, and in the ethiodized oil deposit area after TACE.

### Statistical analysis and propensity score matching

Survival was measured from the date of C-ion RT or TACE initiation to the date of death or the most recent follow-up. LC was defined as no evidence of local recurrence. Progression-free survival (PFS) was measured from the initiation of C-ion RT or TACE to the date of the first tumor progression or death from any cause. Probabilities of OS, LC, and PFS rates were calculated using the Kaplan-Meier method, and a log-rank test was used to compare between 2 survival curves for univariate analyses. A Mann-Whitney U test was used for statistical analysis of differences in patient characteristics. A chi-squared test with Yates’ continuity correction and a two-tailed Fisher’s exact test were used to compare categorical data and to test for differences of progression according to Child-Pugh class between C-ion RT and TACE.

PSM was performed using binary logistic regression to generate a propensity score for each patient. The variables comprehensively selected for propensity score generation included age, sex, performance status, tumor size, Child-Pugh class, BCLC, and alpha-fetoprotein. Subsequently, a one-to-one nearest-neighbor match between patients treated with C-ion RT and TACE was obtained.

The statistical tests were two-sided, and *p* < 0.05 was considered statistically significant. All statistical analyses were performed using Statistical Package for the Social Sciences software, version 25.0 (IBM Inc., Armonk, NY, USA).

## Results

### Characteristics of all eligible patients

Of 124 HCC patients who received C-ion RT during the study period, 53 patients had single HCC and of these, 31 patients had received C-ion RT as a primary treatment. The 31 patients who received C-ion RT were enrolled in this study. Among 353 HCC patients who received TACE, 102 patients had single HCC of these, 29 patients received TACE as a primary treatment, and 6 patients with insufficient clinical data were excluded. Therefore, 23 patients who received TACE were enrolled in this study. The baseline demographics of the patients are shown in Table 1.

**Table 1:** Characteristics of all analyzed patients

	C-ion RT (*n* = 31)	TACE (*n* = 23)	*p-*value
Age, year, median (range)	78 (45–95)	76 (59–90)	0.35
Sex (male: female)	15:16	10:13	0.73
AFP, ng/ml, median (range)	11.3 (1.6–28,006)	9.1 (2.0–300.4)	0.23
PS (0:1:2)	18:11:2	12:11:0	0.90
Child-Pugh class (A:B:C)	29:2:0	14:9:0	< 0.01
BCLC classification (A:B:C)	18:0:13	13:0:10	0.91
Tumor size, mm, median (range)	34 (11–78)	27 (8–60)	< 0.05
Etiology, (HCV-Ag:HBs-Ab:NASH/NAFLD:Alcohol)	19:5:6:1	15:1:5:2	
Total dose of C-ion RT, (52.8 Gy (RBE)/4 fr: 60 Gy (RBE)/4 fr: 60 Gy (RBE)/12 fr)	16:14:1		

*Abbreviations: AFP* alpha-fetoprotein, *BCLC* Barcelona Clinic Liver Cancer, *C-ion RT* carbon ion radiotherapy, *fr* fractions, *HBs-Ab* hepatitis B surface antibody, *HCV-Ag* hepatitis C antigen, *NASH/NAFLD* non-alcoholic steatohepatitis/non-alcoholic fatty liver disease, *PS* performance status, *RBE* relative biologic effectiveness, *TACE* transarterial chemoembolization

### Clinical outcomes of all eligible patients

**Fig. 2:**
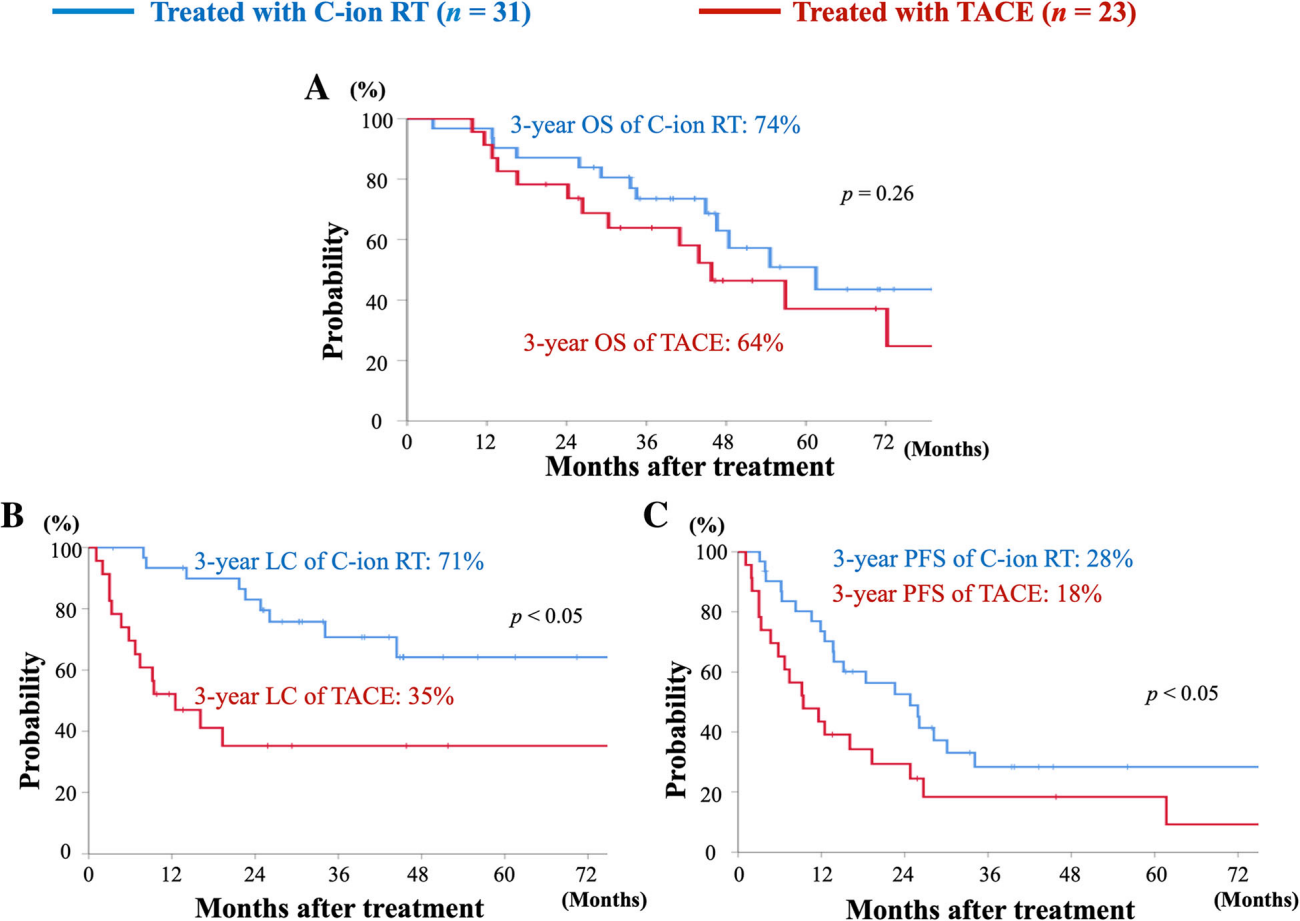
Survival curves comparing C-ion RT and TACE for all analyzed patients. **a** Overall survival curves for C-ion RT (blue) and TACE (red). **b** Local control curves for C-ion RT (blue) and TACE (red). **c** Progression-free survival curves for C-ion RT (blue) and TACE (red)

In all eligible patients, survival curves of C-ion RT and TACE are shown in Fig. 2. The median follow-up durations for C-ion RT and TACE were 43 (range, 4–84) and 37 (range, 10–114) months, respectively. The estimated 3-year OS, LC, and PFS rates in the C-ion RT versus TACE groups were 74% versus 64% (*p* = 0.26), 71% versus 35% (*p* < 0.05), and 28% versus 18% (*p* < 0.05), respectively.

Recurrence after C-ion RT was observed in 19 patients; 4 patients had local recurrence, 14 patients had intrahepatic recurrence outside the target region, and 1 patient had distant metastases to the adrenal grand. Of the 4 patients with local recurrence, 1 patient received C-ion RT as re-irradiation and 3 patients received TACE. Of the 14 patients with intrahepatic recurrence outside the target region, 12 patients received TACE, 1 patient received RFA, and 1 patient received hepatic arterial infusion chemotherapy. One patient with distant metastases received TACE.

Recurrence after TACE was observed in 20 patients; 9 patients had local recurrence, 5 patients had both local recurrence and intrahepatic recurrence outside the target region, and 6 patients had intrahepatic recurrence outside the target region. Of the 9 patients with local recurrence, 5 patients received TACE and 2 patients received RFA. All 5 patients with both local recurrence and intrahepatic recurrence outside the target region received TACE. Of 6 patients with intrahepatic recurrence outside the target region, 4 patients received TACE, and 1 patient received RFA.

With regard to liver function within 3 months from the initiation of treatment, 2 of 29 patients with Child-Pugh class A progressed to class B after C-ion RT. After TACE, 5 of 14 patients with Child-Pugh class A progressed to class B, and 1 of 14 patients with Child-Pugh class A progressed to class C (Table 2). The number of patients who progressed to a worse Child-Pugh class was significantly higher in the TACE group than in the C-ion RT group (*p* = 0.11).

**Table 2:** Child-Pugh class before and after treatment in all analyzed patients

Progression of Child-Pugh class before and after treatment	C-ion RT (*n* = 31)	TACE (*n* = 23)
A. Within 3 months from treatment initiation		
A to A	27	8
A to B	2	5
A to C	0	1
B to B	2	9
B to C	0	0
B At three months after initiation of treatment		
A to A	27	11
A to B	2	2
A to C	0	1
B to B	2	9
B to C	0	0

*Abbreviations: C-ion RT* carbon ion radiotherapy, *TACE* transarterial chemoembolization

After 3 months from the initiation of treatment, 2 of 29 patients with Child-Pugh class A progressed to class B or class C after C-ion RT. After TACE, 2 of 14 patients with Child-Pugh class A progressed to class B, and 1 of 14 patients with Child-Pugh class A progressed to class C (Table 2). There were no significant differences in the number of patients who progressed to a worse Child-Pugh class between the C-ion RT and TACE groups (*p* = 0.73).

### Characteristics of patients after propensity score matching

Seventeen matched pairs of patients from each treatment group were identified. Patient characteristics after PSM are shown in Table 3.

**Table 3:** Characteristics of patients selected after propensity score matching

	C-ion RT (*n* = 17)	TACE (*n* = 17)	*p-*value
Age, year, median (range)	75 (45–85)	78 (59–90)	0.93
Sex ratio (male: female)	8:9	9:8	0.74
AFP, ng/ml, median (range)	8.8 (1.6–386.2)	8.0 (2.0–175.6)	1.00
PS (0:1:2)	8:7:2	8:9:0	0.72
Child-Pugh class (A:B:C)	15:2:0	14:3:0	0.47
BCLC classification (A:B:C)	8:0:9	9:0:8	0.74
Tumor size, mm, median (range)	30 (11–64)	30 (8–60)	0.98
Etiology, (HCV-Ag:HBs-Ab:NASH/NAFLD:Alcohol)	11:2:4:0	10:1:4:2	
Total dose of C-ion RT, (52.8 Gy (RBE)/4 fr: 60 Gy (RBE)/4 fr: 60 Gy (RBE)/12 fr)	7:10:0		

*Abbreviations: AFP* alpha-fetoprotein, *BCLC* Barcelona Clinic Liver Cancer, *C-ion RT* carbon ion radiotherapy, *fr* fractions, *HBs-Ab* hepatitis B surface antibody, *HCV-Ag* hepatitis C antigen, *NASH/NAFLD* non-alcoholic steatohepatitis/non-alcoholic fatty liver disease, *PS* performance status, *RBE* relative biologic effectiveness, *TACE* transarterial chemoembolization

### Clinical outcomes after propensity score matching

**Fig. 3:**
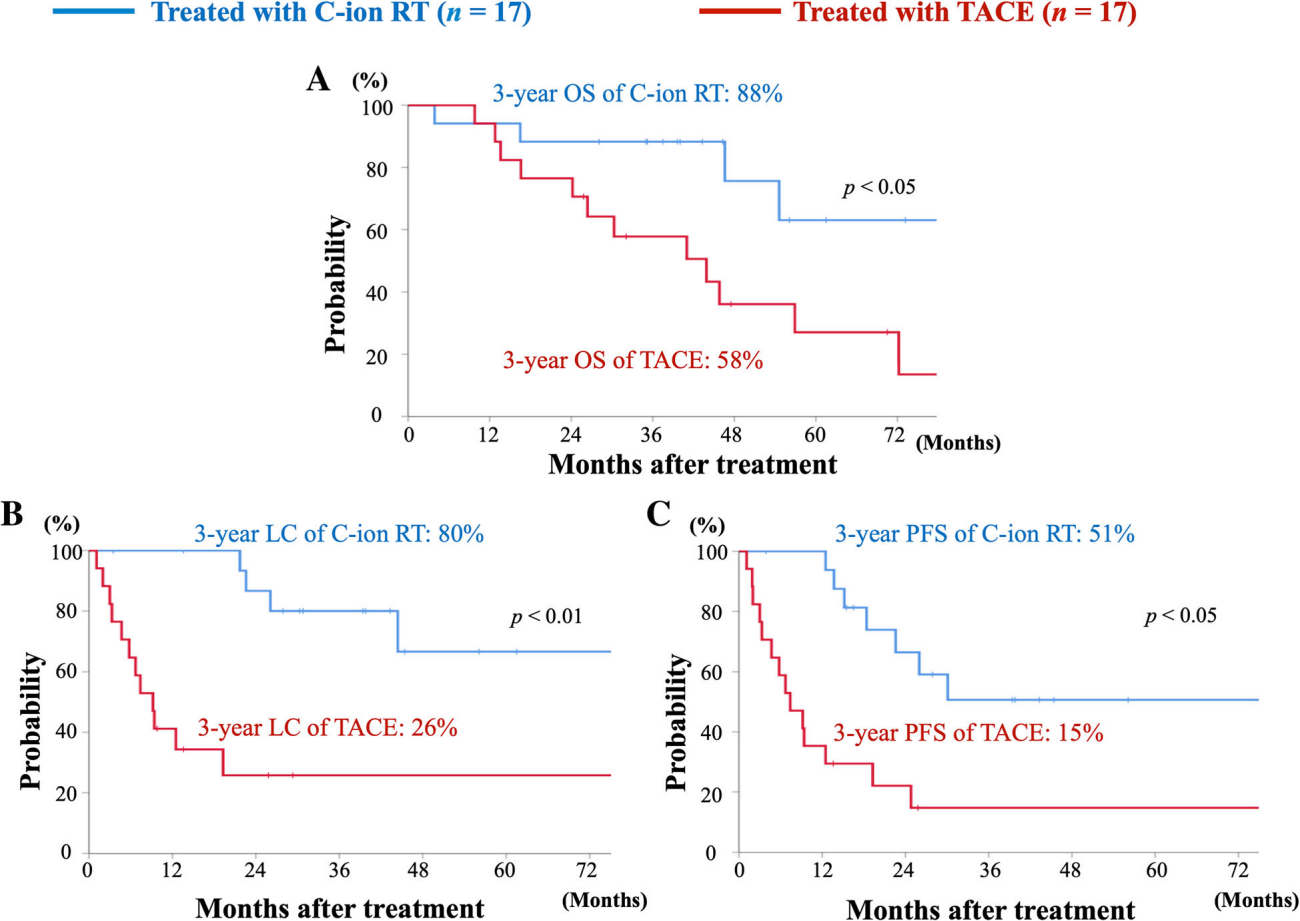
Survival curves comparing C-ion RT and TACE after PSM. **a** Overall survival curves for C-ion RT (blue) and TACE (red). **b** Local control curves for C-ion RT (blue) and TACE (red). **c** Progression-free survival curves for C-ion RT (blue) and TACE (red)

Survival curves of C-ion RT and TACE are shown in Fig. 3. The median follow-up durations in C-ion RT and TACE were 43 (range, 4–84) months and 32 (range, 10–114) months, respectively. The estimated 3-year OS, LC, and PFS rates in C-ion RT versus TACE were 88% versus 58% (*p* < 0.05), 80% versus 26% (*p* < 0.01), and 51% versus 15% (*p* < 0.05), respectively.

Recurrence after C-ion RT was observed in 7 patients; 2 patients had local recurrence and 5 patients had intrahepatic recurrence outside the target region. Of 7 patients with local recurrence, 2 patients received TACE. All 5 patients with intrahepatic recurrence outside the target region received TACE. The median duration of survival after salvage TACE for intrahepatic recurrence was 33 (range: 2–63) months.

Recurrence after TACE was observed in 15 patients; 9 patients had local recurrence, 4 patients had both local recurrence and intrahepatic recurrence outside the target region, and 2 patients had intrahepatic recurrence outside the target region. Of the 9 patients with local recurrence, 5 patients received TACE and 2 patients received RFA. All 4 patients with both local recurrence and intrahepatic recurrence outside the target region received TACE. The 2 patients with intrahepatic recurrence outside the target region received TACE. The median duration of survival after salvage TACE for intrahepatic recurrence was 26 (range: 6–70) months.

With regard to liver function within 3 months from the initiation of treatment, none of the 15 patients with Child-Pugh class A progressed to class B after C-ion RT, while 5 of 14 patients with Child-Pugh class A progressed to class B, and 1 of 14 patients with Child-Pugh class A progressed to class C after TACE (Table 4). The number of patients who progressed to a worse Child-Pugh class was significantly higher in the TACE group than in the C-ion RT group (*p* < 0.01).

**Table 4:** Child-Pugh class before and after treatment in patients selected after propensity score matching

Progression of Child-Pugh class before and after treatment	C-ion RT (*n* = 17)	TACE (*n* = 17)
A. Within 3 months of treatment initiation		
A to A	15	8
A to B	0	5
A to C	0	1
B to B	2	3
B to C	0	0
B At three months after initiation of treatment		
A to A	15	11
A to B	0	2
A to C	0	1
B to B	2	3
*B to C*	*0*	*0*

*Abbreviations: C-ion RT* carbon ion radiotherapy, *TACE* transarterial chemoembolization

After 3 months, none of the 15 patients with a Child-Pugh class A progressed to class B after C-ion RT, while 2 of 14 patients with Child-Pugh class A progressed to class B, and 1 of 14 patients with Child-Pugh class A progressed to class C after TACE (Table 4). There were no significant differences in the number of patients who progressed to a worse Child-Pugh class between the C-ion RT and TACE groups (*p* = 0.23).

## Discussion

This is the first study comparing the clinical outcomes of C-ion RT and TACE for single HCC as a primary treatment after matching patient characteristics utilizing PSM. In our study, the 3-year OS, LC, and PFS rates in C-ion RT versus TACE were 88% versus 58% (*p* < 0.05), 80% versus 26% (*p* < 0.01), and 51% versus 15% (*p* < 0.05), after PSM. Our study showed more favorable clinical outcomes for C-ion RT than for TACE in relation to single HCC as a primary treatment.

Previous studies involving C-ion RT have shown encouraging clinical outcomes that indicate C-ion RT would be an alternative treatment option for patients for which surgery or RFA are not viable options [12–17, 29]. Shibuya et al. demonstrated clinical outcomes of C-ion RT for HCC in a multi-institutional retrospective analysis and reported 2-year OS and LC rates for single HCC of 84 and 87%, respectively [13]. In terms of C-ion RT toxicity, Kasuya et al. showed clinical outcomes of C-ion RT for HCC in prospective trials, and reported that 7 and 6% of patients showed Child-Pugh class progression in the acute and late phases, respectively [15]. In the present study, after utilizing PSM, no patients showed Child-Pugh class progression in C-ion RT. Two other studies have assessed hypofractionated C-ion RT (e.g. ≤12 fractions) [12, 16]. Thus, similar efficacy and toxicity results were obtained in our study compared to previous clinical results concerning C-ion RT for HCC using a similar approach that included dose fractionations and target volumes.

This study did not include the patients with BCLC stage B HCC. TACE is the standard treatment for patients with BCLC stage B HCC, according to various guidelines [1, 11]. However, there are some patients who are ineligible for surgery or who decline surgery and/or RFA, even in BCLC stage A. In a retrospective study, Terzi et al. reported that the 3-year OS rate was 50% for 148 patients with single HCC treated with TACE [6]. Kudo et al. reported a follow-up survey of HCC, and the 3-year OS was 58% for 6069 patients with single HCC treated with TACE [3]. These TACE results for single HCC appear comparable to the clinical outcomes of TACE found in the present study. Moreover, 2-year LC rates of TACE have been found to range from 28 to 41%, which is similar to the LC rate range in our study [4–9]. When comparing clinical outcomes of C-ion RT and TACE, our study showed that C-ion RT had higher LC rates.

Several techniques of TACE have been employed for treating HCC. Scheduled repetition of TACE is one of the techniques [30]. However, none of the patients in our cohort received scheduled repeating TACE. Notably, several researchers have reported favorable clinical outcomes in patients who received a single session of super selective TACE, including those with tumors of 50 mm or larger [8, 31]; super selective TACE is considered indispensable in maximizing the control of targeted tumors with minimal liver toxicities [8, 31, 32]. Although a single session of standard TACE may not be adequate for lesions of 30 mm or larger, a single session of super selective TACE offers higher treatment benefits, and was therefore employed in the present study with the aim of achieving local control.

Liver function status is one of the prognostic factors for OS [9, 33]. In the present study, patients who had undergone TACE more frequently progressed to a significantly worse Child-Pugh class within 3 months. Repeated TACE was performed in 79% of patients with Child-Pugh class A after TACE, while 41% of patients received TACE after C-ion RT as a second treatment. Llovet et al. reported that patients treated with multiple courses of TACE showed Child-Pugh class progression more often than patients treated with a single TACE course [9]. The greater potential likelihood of LC with C-ion RT can reduce or delay subsequent treatments for intrahepatic recurrence. In addition, liver function preservation with C-ion RT may also contribute to a favorable OS.

Prospective randomized controlled trials are ideal to compare the effectiveness of different treatment modalities. However, there have been no reports concerning clinical benefits evaluated through directly comparing C-ion RT and other cancer treatment modalities for HCC. To address these limitations, we aimed to compare clinical outcomes of 477 HCCs treated with C-ion RT or TACE. After matching patient characteristics using PSM, C-ion RT showed more favorable clinical outcomes compared to that of TACE for OS, LC, PFS, and toxicity levels. The relatively small number of matched patients in our study indicated that patient- and disease-related factors varied in clinical practice and that future multi-institutional studies with larger cohorts, such as registry data, are needed to confirm our findings.

This study had several limitations. First, the present study was a single institutional analysis with a small number of matched patients, as described above. Second, the retrospective nature of the study, with a limited follow-up period, may have led to potential biases related to unobserved confounders (i.e., unmeasured patient selection factors for clinical endpoints), which could have remained despite careful matching utilizing PSM. Third, there may have been an economic bias between the C-ion RT and TACE groups, since the cost of C-ion RT is higher than that of TACE.

## Conclusions

In summary, our study results indicate that C-ion RT showed more favorable clinical outcomes than did TACE for single HCC patients as a primary treatment; there have been no previous comparative studies reporting on clinical outcomes between C-ion RT and TACE for single HCC as a primary treatment utilizing propensity score matching. However, it would be premature to conclude that C-ion RT was superior because of the small number of matched patients from a single institution investigated in this study. Therefore, further studies are required to evaluate the effectiveness of both treatment modalities in patients with single HCC who are ineligible for surgery and/or RFA.

## Data Availability

The datasets generated and/or analysed during the current study are not publicly available because they contain personal information but are available from the corresponding author on reasonable request.

## Abbreviations

BCLC: Barcelona clinic liver cancer
C-ion RT: Carbon ion radiotherapy
CT: Computed tomography
CTV: Clinical target volume
GTV: Gross tumor volume
HCC: Hepatocellular carcinoma
MRI: Magnetic resonance imaging
PFS: Progression-free survival
PSM: Propensity score matching
PTV: Planning target volume
RBE: Relative biologic effectiveness
RFA: Radiofrequency ablation
TACE: Transarterial chemoembolization
